# Experimental Induction of Extreme Indented Growth Rings (Hazel Wood) in *Pinus halepensis* Miller by Wide and Long Parallel Bark and Vascular Cambium Woundings

**DOI:** 10.3390/plants13162265

**Published:** 2024-08-15

**Authors:** Simcha Lev-Yadun, Ján Kováč, Jaroslav Ďurkovič, Vladimír Račko

**Affiliations:** 1 Department of Biology & Environment, Faculty of Natural Sciences, University of Haifa—Oranim, Tivon 36006, Israel; 2 Department of Phytology, Technical University in Zvolen, 96001 Zvolen, Slovakia; jaroslav.durkovic@tuzvo.sk; 3 Department of Wood Science, Technical University in Zvolen, 96001 Zvolen, Slovakia; racko@tuzvo.sk

**Keywords:** figured wood, hazelwood, indented growth rings, *Pinus*, vascular cambium, wounding

## Abstract

Indented growth rings were found long ago to be experimentally induced in *Pinus halepensis* Miller by thin parallel axial scratching of the bark up to the vascular cambium with a sharp blade. Here, we show that when the bark and vascular cambium of *P*. *halepensis* are wounded by wide and long parallel axial wounds (“windows”) rather than by thin scratches, the induced indented growth rings become dramatically more indented. All ten trees that were wounded by long parallel “windows” responded with very strong growth (especially in the first two years) that resulted in the formation of very conspicuous, extremely indented growth rings in the wood formed in between the long and wide woundings. This is true for both the trunks that were wounded all around their circumference and those that were wounded only in part of their circumference. We also suggest further lines of research.

## 1. Introduction

Indented growth rings, commonly known as “hazel wood”, is a rare, abnormal type of figured wood development in which the growth ring borders are wavy [1,2]. Indented growth rings are known to be induced externally under the influence of various environmental factors on cambial wounds, such as fire, rockfalls, and animal and pathogen activity, and also because of unknown reasons, as reviewed in [2]. The formation of indented growth rings in *Pinus halepensis* and other conifers because of an unknown type of induction agent may continue for decades [2]. Wood with indented growth rings is considered to be more decorative than normal wood and may also have better acoustic qualities, making such wood especially suitable for certain types of musical instruments [3,4,5,6]. Indented growth-ring mutations exist in *Cryptomeria japonica* D. Don, and because of their high economic value they were propagated by cuttings [7].

Because of its rarity in nature, it is usually difficult to study the formation of indented growth rings, but the possibility of experimental induction of them by bark and cambial scratching, as was shown in the Mediterranean conifer *Pinus halepensis* [2,8,9], opens new horizons for studying this fascinating and economically valuable phenomenon. It was proposed that such wounding resulted in disturbances of the hormonal balance and polar auxin transport in the cambial region [8].

Lev-Yadun et al. [2] in their review discussed the inherent difficulties in experimentally inducing and studying indented growth rings. (1) Significantly indented growth ring formation cannot be studied in tree seedlings, and some of the questions concerning indented growth rings, especially those related to commercial products (timber, veneer, producing musical instruments), require long-term experiments. Such experiments last not less than 4–10 years and even much longer, in order to produce enough wood with indented growth rings for the manufacture of considerable amounts of figured veneer or commercial figured timber. (2) Such long-term experiments can frequently end with no results because of forest fires, tree death due to insect or fungal attacks, drought, or because of anthropogenic activities such as tree logging, road construction, or building activity. (3) The duration of M.Sc., Ph.D., post-doc, or regular research grants is too short for some of the studies of indented growth ring formation and utilization. For instance, if the basic experiments in inducing extremely indented growth rings are at least 3–4 years long, then, if something else should be examined in addition to the results of the initial experiments, it makes the study last at least 6–8 years.

After it became clear that indented growth rings can be induced in *P. halepensis* by thin parallel axial scratches through the bark up to the vascular cambium with a strong, sharp blade [8,9], the question of the potential of inducing another type or even more extremely indented growth rings by another type of wounding was addressed experimentally. Here, we present the results.

## 2. Results

**Figure 1:**
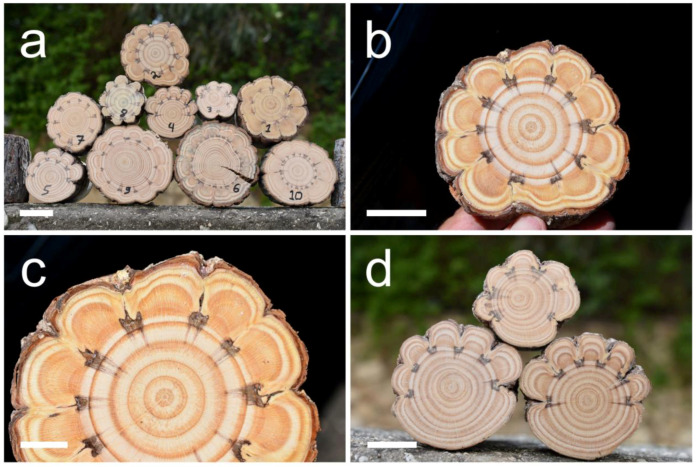
Cross-sections of *Pinus halepensis* trunks in which extremely indented growth-rings were induced by “window” woundings. (**a**) A pile of the ten trunks showing extremely indented growth rings. The numbers written on the cross-sections are the tree numbers that appear in Table 1. The diameter of the cross-section of tree number 1 is 97 mm. (**b**) A full view of the extremely indented growth rings in the trunk of tree number two. (**c**) A close-up of the extremely indented growth rings in the trunk of tree number two. (**d**) The three trunks (numbers 3–5) in which the “window” woundings were made in only part of the circumference. Scale bars: (**a**) = 50 mm, (**b**,**d**) = 30 mm, (**c**) = 15 mm.

All ten trees that were wounded by long “windows” responded with very strong growth that resulted in the formation of very conspicuous, extremely indented growth rings in the wood formed between the long parallel woundings (Figure 1a–c). This is true for both the trunks that were wounded all around their circumference (trees number 1–2, 6–10) and those that were wounded only in part of their circumference (trees number 3–5) (Figure 1d).

**Figure 2:**
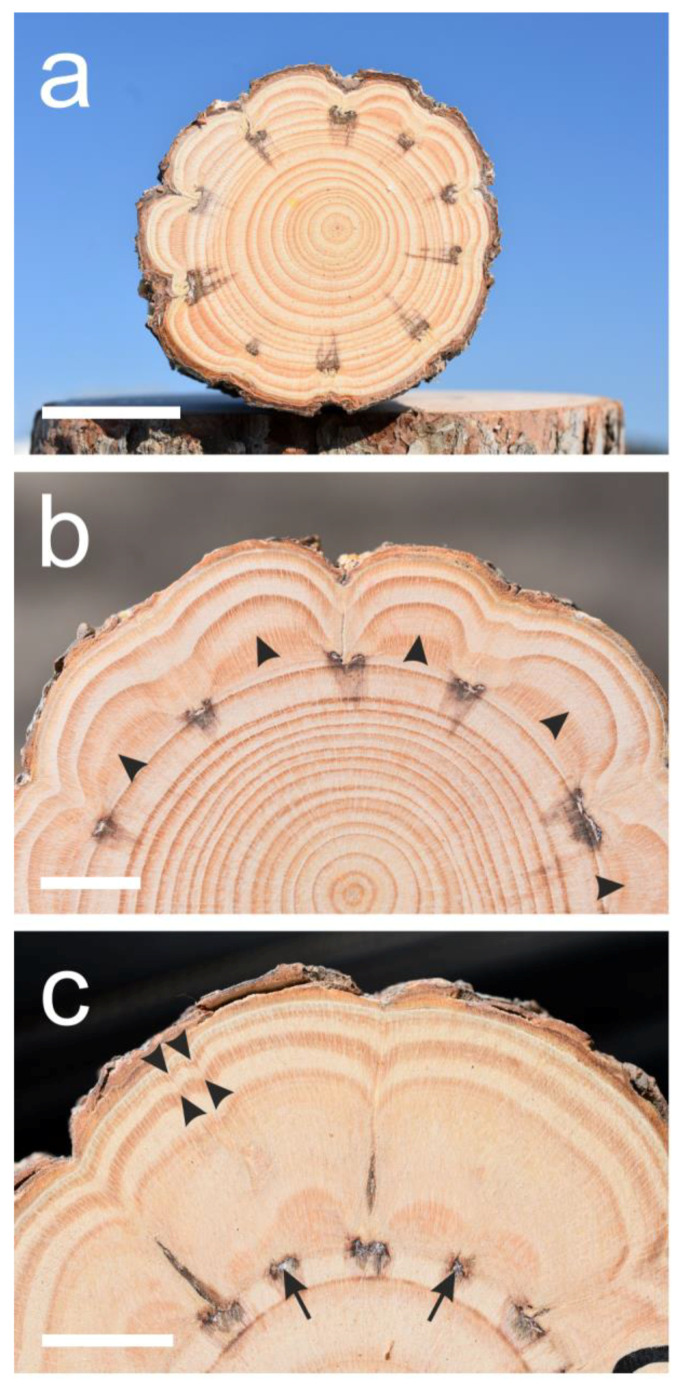
Cross-sections of suppressed *Pinus halepensis* trees. (**a**) Suppressed tree number seven. Not all ten “window” woundings induced the extremely indented growth rings. (**b**) A sector of the cross-section of tree number six showing the non-sharp first growth ring border (arrowheads) formed after the wounding. (**c**) A sector of the cross-section of tree number ten that was wounded both by “window” and thin scratches (arrows) between them. The thin scratches caused some indentations (arrowheads) but no clear growth ring borders in the first year. Scale bars: (**a**) = 30 mm, (**b**,**c**) = 15 mm.

The woundings induced extremely indented growth rings only where they occurred, and the strong influence on the growth rings stopped within a few milimeters from the upper and lower edge of the wounding. However, in some of the trunks, some of the growth rings below the wounded sector continued to be wider and somewhat indented for several centimeters, although not forming clearly indented growth rings, but we did not pursue this effect in depth. The only conspicuous partial exception is tree number seven, which was a suppressed tree that grew in the Hulda forest in the shade of much taller *Pinus halepensis* trees. In that suppressed tree, four out of the ten woundings resulted in a very mild response, and the responses in the other six woundings were not as strong as in the other trees (Figure 2a).

Tree number eight died three years after the wounding for an unknown reason, but demonstrated an extreme growth-ring indentation (Figure 1a). As can be seen in Figure 1 and Figure 2, the first and second growth rings formed after the wounding were the widest (12.4 mm on average for the first two years together versus 5.7 mm for the last two years together), and after the second year, the width of the outer growth rings, even when extremely indented, became narrower. The growth ring border of the first year formed some months after the wounding was not as clear and not as sharp (Figure 2b) as the other growth ring borders. In the two trees (trees number 6 and 10) that were wounded by thin scratches in between the “windows”, growth-ring indentation was induced by the thin scratches only in the first year, but the growth ring border was not clear and the influence declined after the first year (Figure 2c).

## 3. Discussion

Very few studies have shown or discussed the experimental induction of indented growth rings in conifers [2,8,9]. Therefore, we know very little about their experimental induction. After we managed to regularly induce classic indented growth rings in *Pinus halepensis* by thin and long parallel scratches of the cambium [8,9], we decided to experiment with broader parallel “window” woundings. Our initial hypothesis was that the regenerating vascular cambial strips from the flanks of the “windows” would meet after several years at the center of each “window” and with the passing years would form many parallel narrow bands of indented growth rings of the type found in wound closure in many tree species ([2] and the citations therein). We were surprised to find that the type of indented growth rings we induced in our experiment was very different. When tree number one in the Hulda forest had to be cut only 13 months after the wounding because of bulldozer activity to broaden a road, the extremeness of the induced indented growth-rings compared to those induced by narrow cambial scratches was a surprise, but at that stage we still did not know if it was a regular phenomenon in *Pinus halepensis* or something related to an unknown pathogenic reaction. When we sampled tree number two (which grew in the Ben-Shemen forest) after 40 months, 8 months before the planned sampling because of a spreading forest fire, we realized that this strong cambial reaction is the rule for such wounding.

The strong cambial activity in the sectors between the “windows” of partial girdling causing the extreme indented growth rings that we show seems to be the synergistic influence of at least four factors: (1) direct wound responses that basically induce indented growth rings [2,8,9]; (2) the influence of partial girdling, which results in increased local cambial activity in the non-girdled sectors of the trunk [10,11,12]; (3) a typical increase in cambial activity and radial growth above bark girdling [13,14] because the many broad woundings cause many local girdling-like effects; and (4) when trees close large wounds in the vascular cambium, the process may take several years, many decades, or even over a century, depending on the size of the wound and the rate of growth [15,16]. The cessation of the production of very wide growth rings after the second growth season from wounding that we found seems to be a typical response to wounding of *Pinus halepensis* [17] (p. 451, Figure 2 therein) and indicates that the considerable damage to the phloem by the wounding caused an effect resembling cambial responses to full girdling.

## 4. Further Research

There are currently two essential additional steps for studying the experimental induction of extremely indented growth rings. The first and obvious one, typical for the practices and methods we use in our labs, is to test the possibility of inducing extremely indented growth rings in several other conifer species. In Israel, the conifer trees species *Pinus brutia* Ten., *Pinus canariensis* C. Sm., *Pinus pinea* L., and *Cupressus sempervirens* L. are currently being studied. In Slovakia, the coniferous tree species *Picea abies* (L.) H. Karst., *Pinus sylvestris* L., *Larix decidua* Mill., and *Abies alba* Mill. are currently under study. These experiments, which started in Israel in the winter of 2023/2024 and in Slovakia in the spring of 2024, are expected to take at least the next four to five years, and they will allow us to better understand if the very strong effect of such wounding on growth rings is common or species-specific. Of course, studying many other conifers and dicots is also a natural continuation of our study. Studying the potential commercial value of the species we study requires additional years of research. In both *Pinus halepensis* and the other species, when the many new samples from the running experiments provide a sufficient amount of samples, they will allow careful anatomical studies and some tests of the possibility to use such treatments in order to obtain figured wood for the industry.

Another essential step, which is outside the practices we use in our labs, is to identify the genes and regulatory factors (hormones and other molecules) involved in the induction and development of extremely indented growth rings. A comparison between typical experimentally induced indented growth rings and extreme ones is also required. The fact that the formation of extremely indented growth rings requires several years makes that step difficult and expensive, because repeated sampling over several years is required. At least our studies have already provided and will continue to provide the experimental systems required for studying the molecular and cell biology aspects of extremely indented growth ring formation. Practically, conifers, for which most or the whole genome was sequenced, for instance *Picea abies* [18], *Picea glauca* (Moench) Voss [19], *Pinus lambertiana* Douglas [20], *Pinus taeda* L. [21], and *Pseudotsuga menziesii* (Mirb.) Franco [22,23], seem to be suitable candidates for such studies.

## 5. Materials and Methods

**Figure 3:**
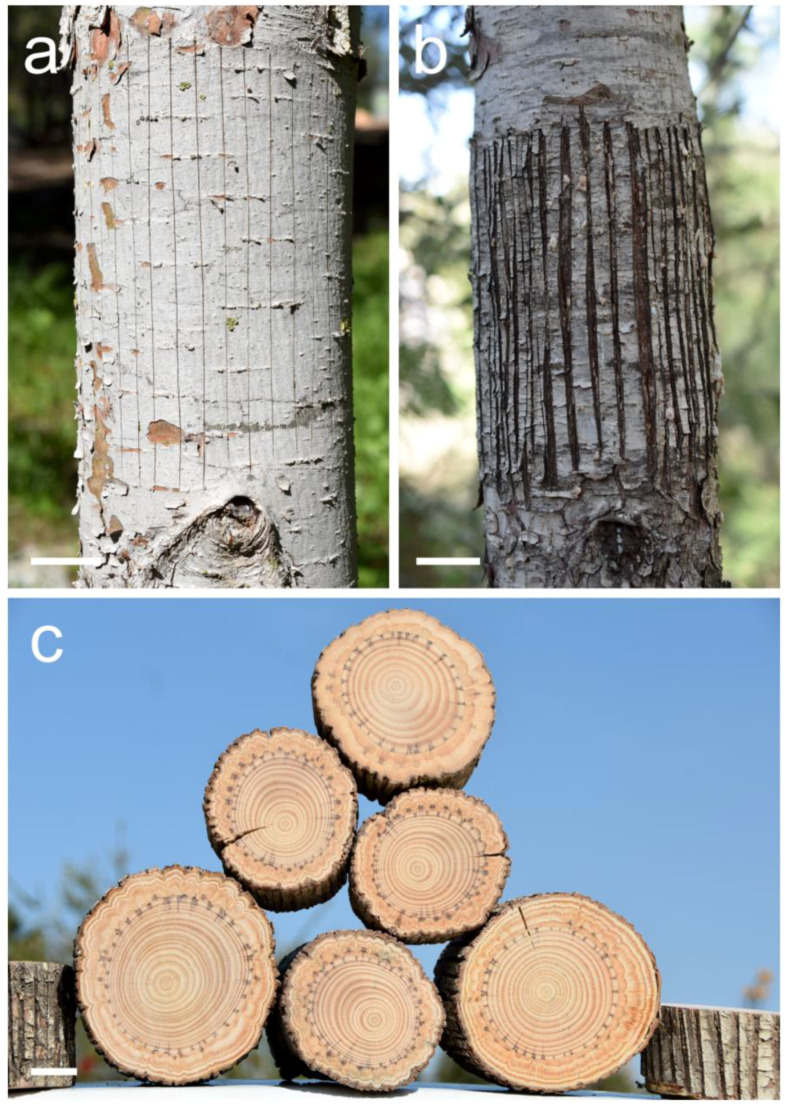
Thin axial scratches in the bark and cambium of a *Pinus halepensis* trunk. (**a**) Fourteen just-made scratches with a sharp blade in order to induce indented growth rings. Photographed February 2024. (**b**) Scratches with a sharp blade in order to induce indented growth rings as they looked after almost four years. Photographed February 2024. (**c**) A pile of six trunks of *Pinus halepensis* in which indented growth rings were induced by thin axial scratches several years earlier. Scale bars: (**a**–**c**) = 30 mm.

**Figure 4:**
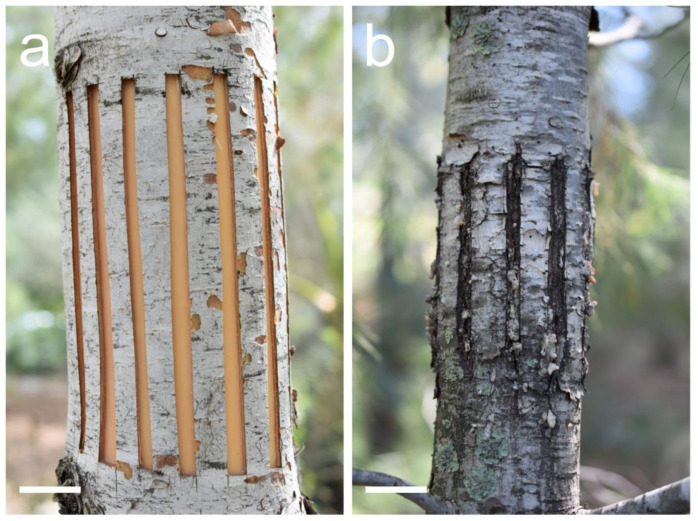
Wide axial scratches that we call “windows” in the bark and cambium of a *Pinus halepensis* trunk. (**a**) Just-made scratches with a sharp blade in order to induce extremely indented growth rings. Photographed February 2024. (**b**) Scratches with a sharp blade, i.e., “windows”, in trunk number seven as they looked after almost four years. Photographed February 2024. Scale bars: (**a**,**b**) = 30 mm.

In our broad and multi-issue study about indented growth ring formation that started in the year 2018, the Mediterranean conifer species *Pinus halepensis* is our basic model. We try and examine various aspects of naturally occurring and experimentally induced indented growth ring formation with this species, and when we manage to progress with an issue, we later examine or experiment with other Mediterranean and European conifer species. After succeeding with regular induction of indented growth rings in this species by thin parellel scratches in the bark all the way to the vascular cambium with the blade of a sharp utility knife (Figure 3a–c) [8,9], we decided to try wider parallel axial cambial woundings, what we call “windows” (Figure 4a,b).

All the trees we used belonged to the naturally seeded progeny of older planted trees that established the forest about six decades earlier. The trees were about five years old when they reached breast height, and for estimating tree age when wounded, this number should be added to the number of growth rings found at the wounding level. Two of the trees grew in the Hulda forest (31°49′55″ N, 34°53′2″ E) and eight in various parts of the Ben-Shemen forest (31°56′54″ N, 34°57′26″ E); both forests are located in central Israel (Table 1). We almost never use very mature trees, because in such trees the bark becomes too thick and hard, not allowing the blades to move in the desired direction, and in some previous experiments with *Pinus halepensis* and other pine species with a thick cork mechanically reenforced by sclereid (stone cell) bands, we realized that the vascular cambium was not always wounded by the blades.

We used the trunks of ten *Pinus halepensis* trees that were mature enough to bear mature seed cones and were several meters tall. The wounded trunk sector was 4–15 years old (the trunk bases were several years older), with only a thin first periderm at the wounded sector. In April 2020 (Table 1), which, under Eastern Mediterranean conditions, is a season of strong cambial activity [24], they were wounded with the blade of a sharp utility knife (6–12 wounds per trunk) at breast height, in the form of ca. 10–15 cm-long and 5–8 mm-wide parallel “windows” (Figure 4a), which, in addition to the wounding, caused repeated partial and sectorial axial girdlings. The length of these “windows” depended on the distance from the branch whorls on the trunks. Seven trees (tree numbers 1–2, 6–10) were wounded by such girdled sectors all around the trunk, and three trees (tree numbers 3–5) were wounded by such girdled sectors only in part of the circumference. In tree numbers six and ten, thin longitudinal scratches with a sharp blade, like those that were used in previous studies [8,9] for the induction of indented growth rings, were made in the middle of the space between each pair of longitudinal “windows” in order to compare the different effects of these two types of bark and cambial wounding.

At the end of the experiment, the trunks were cut with an electric chainsaw and dried for about two months, and then the cross-sections were polished with sandpaper in order to allow for good visualization and photography of the growth rings. Tree number one (Hulda forest) was sampled after only 13 months because of bulldozer activity for preparing a forest road, and tree number two (Ben-Shemen forest) was sampled after only 40 months because of a spreading forest fire.

## Figures and Tables

**Table 1:** Experimental induction of extreme indented growth rings in *Pinus halepensis*.

Tree Number	Date of Wounding	Forest	Age of Wounded Sector (Years)	Date of Sampling	Type of Wounding and Number of Wounds
1	17 April 2020	Hulda	4	17 May 2021	All around (9)
2	19 April 2020	Ben-Shemen	5	23 August 2023	All around (10)
3	19 April 2020	Ben-Shemen	8	30 December 2023	Part (6)
4	19 April 2020	Ben-Shemen	7	30 December 2023	Part (6)
5	19 April 2020	Ben-Shemen	8	30 December 2023	Part (6)
6	19 April 2020	Ben-Shemen	13	1 January 2024	All around (12)
7	17 April 2020	Hulda	13	11 February 2024	All around (10)
8	19 April 2020	Ben-Shemen	5	25 February 2024	All around (9)
9	19 April 2020	Ben-Shemen	15	25 February 2024	All around (11)
10	19 April 2020	Ben-Shemen	5	29 February 2024	All around (9)

## Data Availability

Data are contained within the article.
